# A new genus and species of cyclopoid (Crustacea, Copepoda, Cyclopinidae) from a coastal system in the Gulf of Mexico

**DOI:** 10.3897/zookeys.534.6019

**Published:** 2015-11-11

**Authors:** Eduardo Suárez-Morales, Roberto Javier Almeyda-Artigas

**Affiliations:** 1 El Colegio de la Frontera Sur (ECOSUR), Unidad Chetumal, A.P. 424, Chetumal 77014, Quintana Roo,Mexico; 2 Universidad Autónoma Metropolitana- Xochimilco, Departamento El Hombre y su Ambiente, Mexico, D.F., Mexico

**Keywords:** Free-living copepods, coastal zooplankton, taxonomy, interstitial copepods

## Abstract

A new, monotypic genus of the interstitial marine cyclopoid copepod family Cyclopinidae G.O. Sars, 1913 is described from male and female specimens collected at Laguna de Términos, a large coastal lagoon system in the southern Gulf of Mexico. *Mexiclopina campechana* **gen. et sp. n.** cannot be adequately placed in any extant genus within the family. It differs from other cyclopinid genera in having a unique combination of characters including: 1) absence of modified brush-like seta on the mandibular exopod; 2) maxillule exopod with stout setal elements and brush-like setae absent; 3) basis of mandible with one seta; 4) presence of a modified seta on endopod of fourth leg; 5) fifth leg exopod unsegmented, armed with three elements in the female and five in the male; 6) intercoxal sclerite of first swimming leg with two medial spiniform processes on distal margin. The new genus is monotypic and appears to be most closely related to *Cyclopina* Claus, 1863 and *Heptnerina* Ivanenko & Defaye, 2004; the new species was compared with species of *Cyclopina* and it resembles *Cyclopina americana* Herbst, 1982 and *Cyclopina caissara* Lotufo, 1994. This is the second record of a species of Cyclopinidae in Mexico and the first in the Gulf of Mexico; the number of cyclopinid species recorded from the Americas is now 13.

## Introduction

The cyclopoid copepod family Cyclopinidae G.O. Sars, 1913 is one of the most diverse and successful among the benthic marine poecilostomatoid/cyclopoid copepods. It contains 12 valid genera (Boxshall and Halsey 2004; Boxshall 2015). Members of this family occupy a wide range of habitats, having been reported from shallow coastal environments (Reid 1990; Lotufo 1994; Karanovic 2008), anchialine caves (Jaume and Boxshall 1996a), and deep-sea hydrothermal vents (Ivanenko and Defaye 2004). Its knowledge in the Americas is still developing, but it is clear that its diversity has been more studied in South America than in the other subcontinents (Nicholls 1939; Lotufo and Rocha 1991; Lotufo 1994; Rocha and Botelho1998). Only one species of the diverse and widespread genus *Cyclopina*, *Cyclopina caissara* Lotufo, 1994 has been recorded in Mexico and Central America (Reid 1990; Gómez and Martínez-Arbizu 2004) and nine in South America, mainly in Brazil (Lotufo and Rocha 1991; Lotufo 1994; Rocha and Botelho 1998). Overall, the knowledge of the cyclopinid copepod diversity in this kind of habitats is still lagging and certainly deserves further taxonomic research, particularly in the Northwestern Atlantic region.

Laguna de Términos, in the Mexican state of Campeche, in the southern Gulf of Mexico (between 18°26’ and 18°44’N; 91°13’ and 91°54’W) is one of the largest lagoon estuarine ecosystems of the gulf; it has a significant ecological and economic importance in southeastern Mexico because of its permanent connection to the sea and high productivity and diverse fish fauna (Yáñez-Arancibia and Day 1982; Ramos-Miranda et al. 2006). Copepods have been investigated but only those of the plankton community (Salas-Marmolejo 1981). As part of a study to know the helminth fauna of this coastal system and the role played by invertebrates and vertebrates as intermediate, “transport”, “carrier”, paratenic or definitive hosts, night samples were obtained at shallow areas of the lagoon where a mixture of plankton and epibenthic or interstitial copepods was likely to be collected. Our samples contained a new genus and species of the family Cyclopinidae which is herein fully described and illustrated based on male and female specimens.

## Methods

Night zooplankton samples were obtained on February 13, 2015 with three hand nets (two of 100 and one of 200 µm) in shallow areas (depth: 60–120 cm) of the lagoonal system, particularly at Isla Tortuga (18°44'29.3"N; 91°29'44.6"W). Water temperature was 25 °C, salinity 28psu, and pH slightly alkaline (7.5). Trawls followed a parallel course with respect to the coastline. Samples were placed in a bucket with 5 liters of water; copepods were isolated alive 5 hours after collection, they were later on fixed in 4% formaldehyde buffered with borax (30 g/l of formaldehyde at 40%) and kept in a 5% glycerin/ 70% ethanol solution. More than 35 male and female specimens were taxonomically examined in the laboratory; specimens were processed, dissected and examined following Reid (2003). Dissected specimens/appendages were mounted in semi-permanent slides with glycerine sealed with Entellan®, a commercial, fast drying mounting medium and sealant. Drawings were prepared at 1000× magnification with the aid of a camera lucida mounted on a standard Olympus CX31 compound microscope. Some specimens were prepared for SEM examination with a TOPCON SM-510 microscope at facilities of ECOSUR in Tapachula, Mexico. The process included dehydration of specimens in progressively higher ethanol solutions (60, 70, 80, 96, 100%), critical point drying, and gold-palladium coating (20 nm) following usual methods. This hitherto unknown genus and species was described and illustrated following the current standards for the taxonomic study of the group (Gómez and Martínez Arbizu 2004; Karanovic 2008). The type specimens were deposited in the collection of zooplankton held at El Colegio de la Frontera Sur (ECO-CH-Z), in Chetumal, Mexico and in the National Museum of Natural History, Smithsonian Institution, Washington, D.C. (USNM). Original zooplankton samples containing more non-type specimens remain in the helminth collection of the Universidad Autónoma Metropolitana-Xochimilco, Mexico (CHUX), maintained by the co-author (RJAA).

## Results

### Order Cyclopoida Rafinesque, 1815 Family Cyclopinidae G.O. Sars, 1913

#### Genus Mexiclopina gen. n.

Taxon classificationAnimaliaCyclopoidaCyclopinidae

http://zoobank.org/CE58F654-7286-4B69-9671-9282D5DD69DF

##### Type species.

*Mexiclopina campechana* sp. n.

##### Etymology.

The genus name is composed by the prefix ‘Mexi’ in reference to Mexico, the country from which it was collected and the suffix ‘clopina’ to show its affinity with the genus *Cyclopina*.

##### Diagnosis.

First pedigerous somite free, posterolateral margins of second and third pedigerous somites slightly produced. Caudal ramus with six setae, seta I absent. Female antennule 10-segmented, 6^th^ segment longest; male antennule 15-segmented. Antenna with single exopodal seta. Mandibular palp with one basal seta, 2-segmented endopod and 4-segmented exopod; fourth exopodal segment with two ordinary setae. Maxillule endopod with seven setae, exopod with four short, stout elements. Maxilliped 6-segmented. Legs 1–4 with 3-segmented rami; spine and seta formula as for type species. Endopod segment 3 of leg 4 with modified outer seta. Female fifth leg exopod unsegmented, bearing three elements (setae/spines); male fifth leg exopod unsegmented, armed with five elements, three setae, two spines. Sixth leg with two setae in female, and with two setae plus short spiniform process in male.

#### Mexiclopina campechana sp. n.

Taxon classificationAnimaliaCyclopoidaCyclopinidae

http://zoobank.org/D569F55D-9C92-41E5-842C-C711103C014D

**Figure 1.**
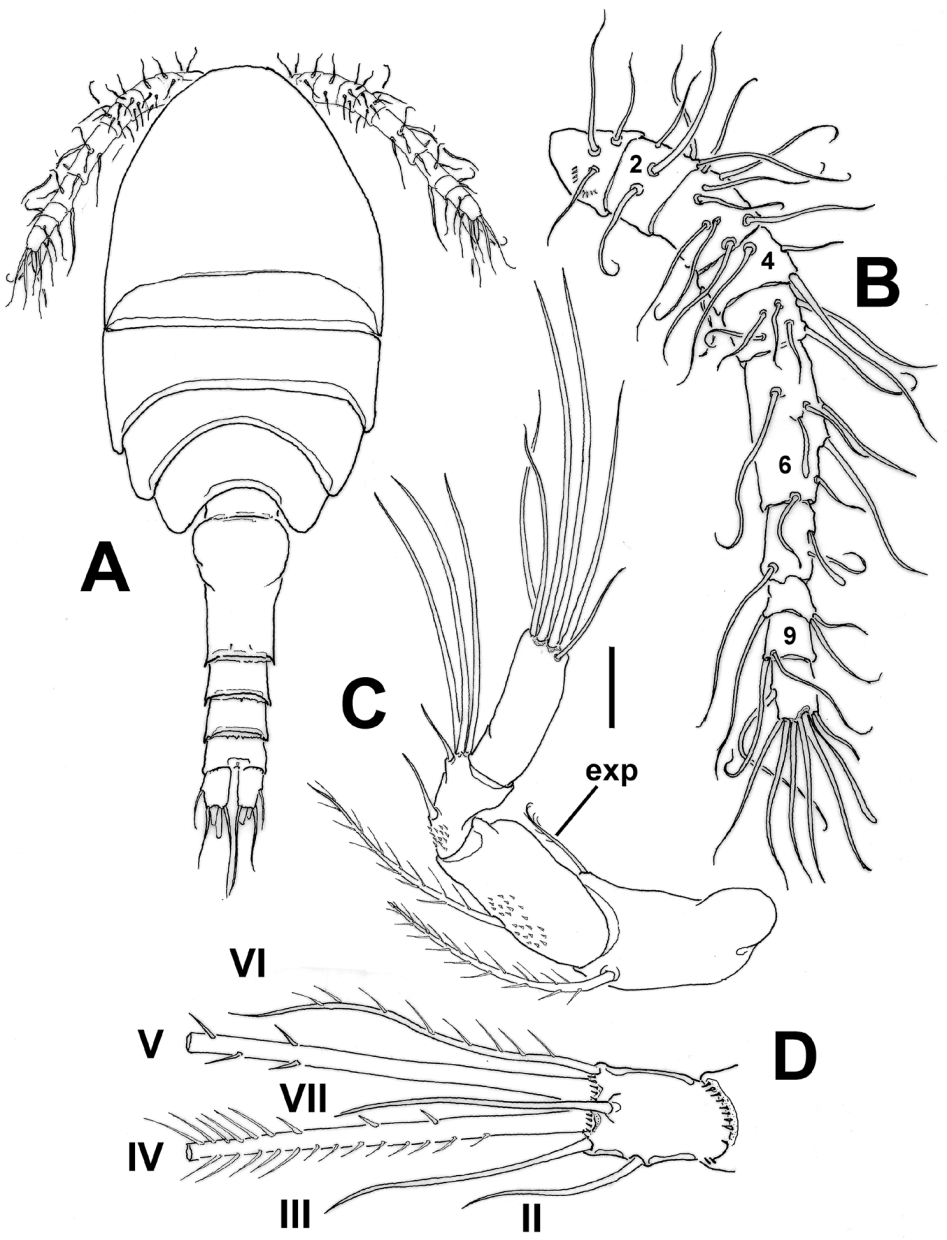
*Mexiclopina campechana* gen. et sp. n., female holotype. **A** habitus in dorsal view **B** antennule **C** antenna **D** left caudal ramus, dorsal view, showing position of caudal setae I-VII (plumosity of setae III and VI not illustrated). Scale bars: 50 µm (**A**); 10 µm (**B–D**).

**Figure 2.**
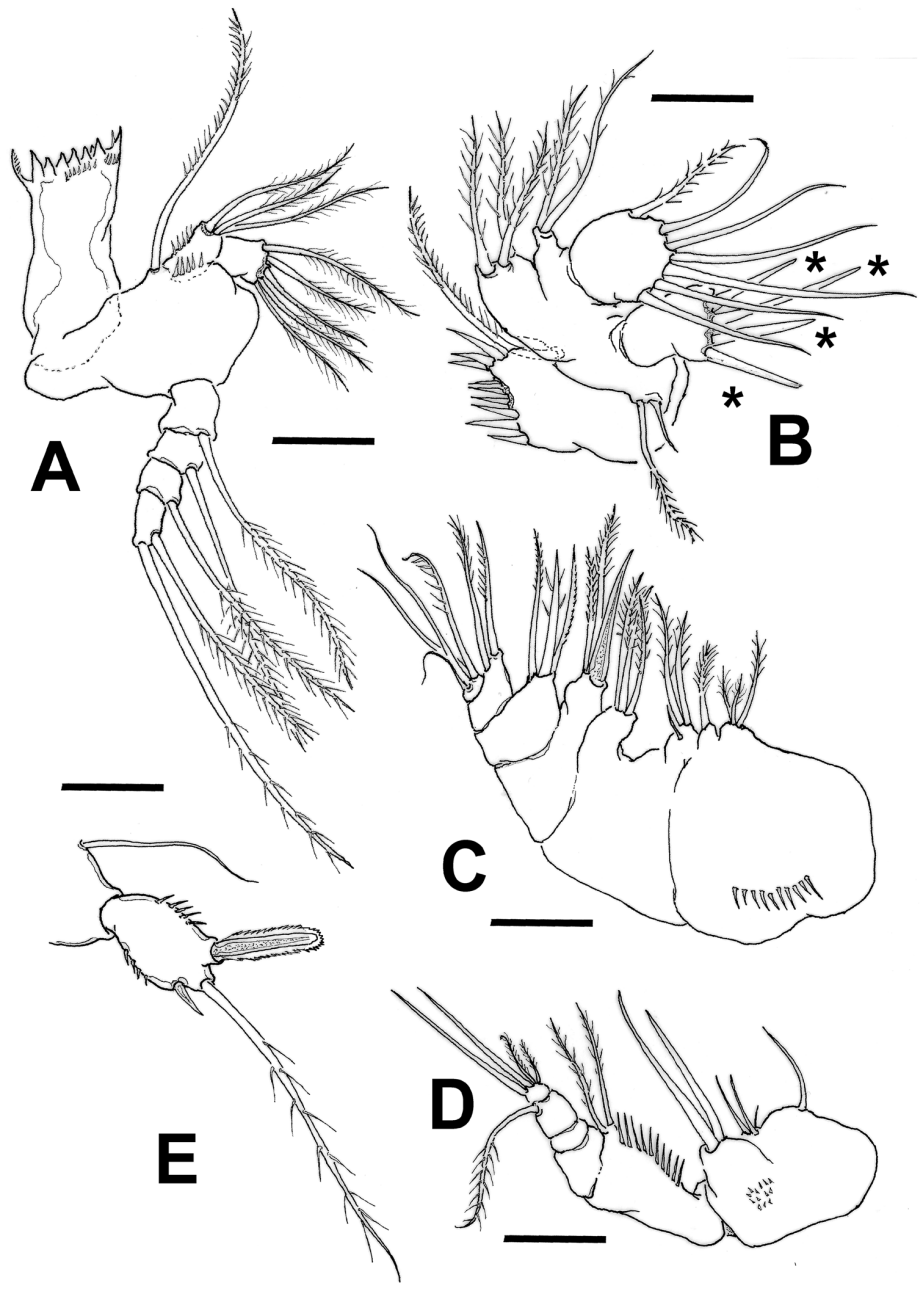
*Mexiclopina campechana* gen. et sp. n., female holotype. **A** mandible **B** maxillule, asterisks indicate stout exopodal setae **C** maxilla **D** maxilliped **E** fifth leg. Scale bars: 10 µm (**A–E**).

**Figure 3.**
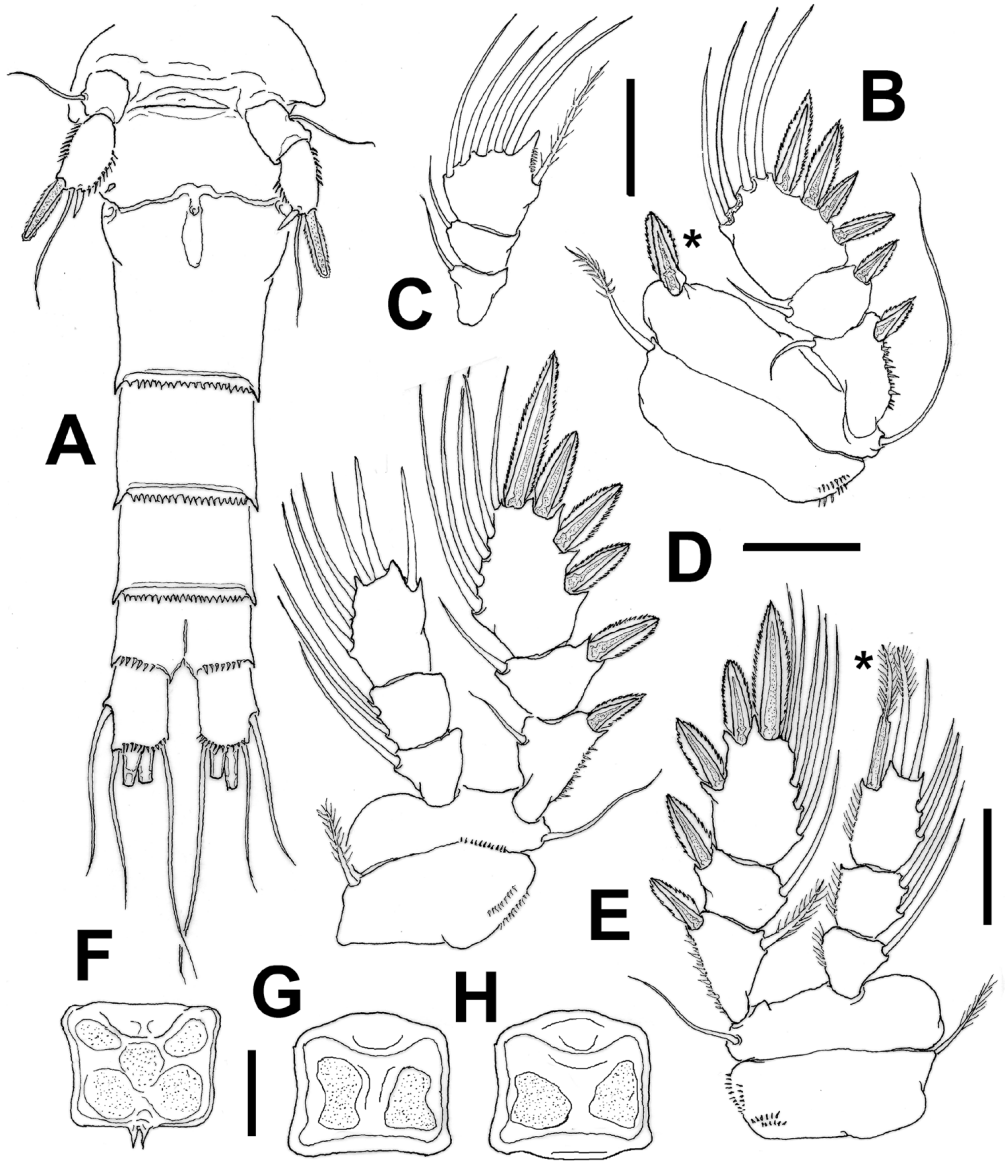
*Mexiclopina campechana* gen. et sp. n., female holotype. **A** urosome showing fifth legs, ventral view **B** first swimming leg with exopod **C** endopod of first leg **D** third leg **E** fourth leg, asterisk indicates modified seta on endopod **F** intercoxal sclerite of first leg showing spiniform processes **G** same, third leg **H** same, fourth leg. Scale bars: 50 µm (**A**); 10 µm (**B–H**).

**Figure 4.**
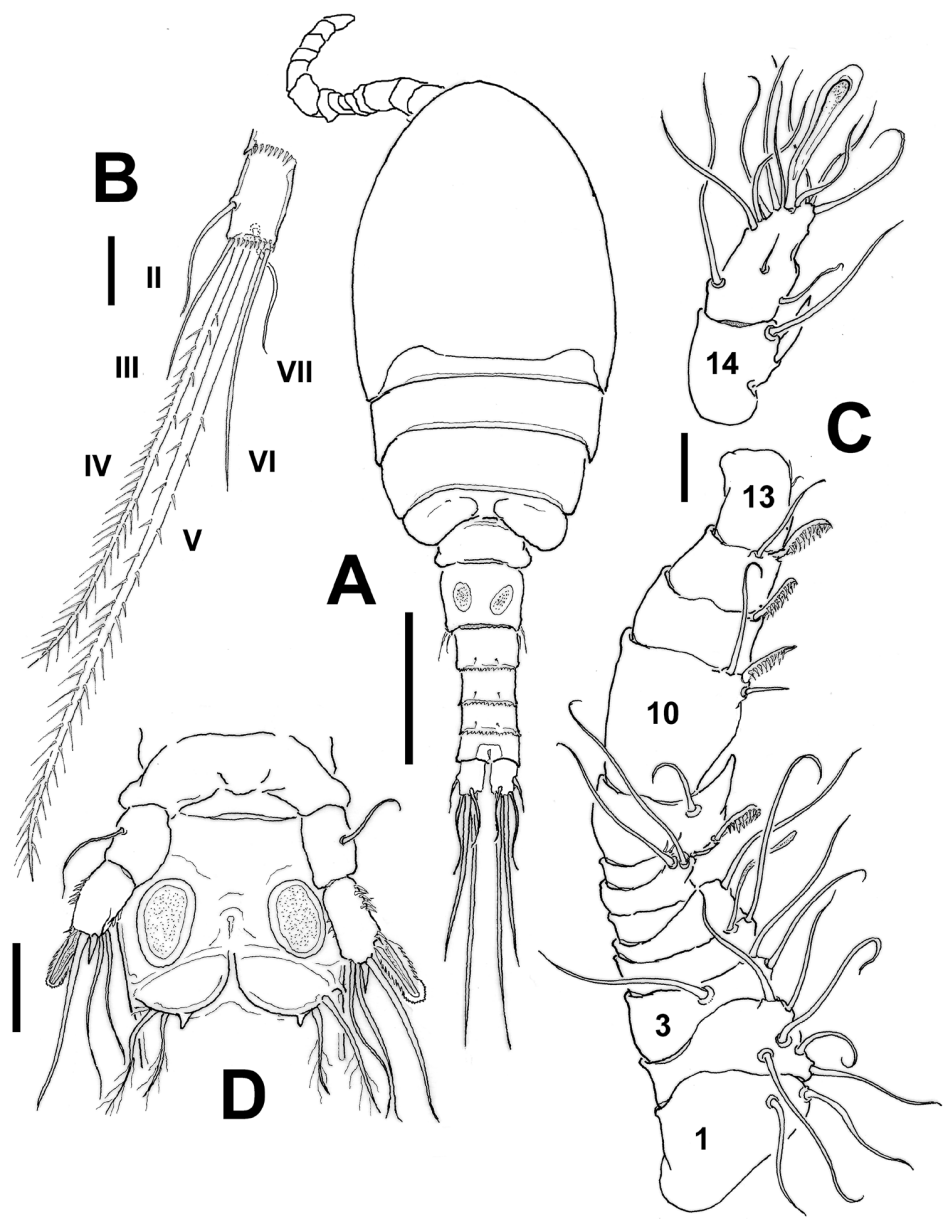
*Mexiclopina campechana* gen. et sp. n., male allotype. **A** habitus, dorsal view **B** right caudal ramus, ventral view showing position of caudal setae I-VII **C** geniculate antennule, segments 14–15 shown separately **D** fifth and sixth legs, ventral view. Scale bars: 50 µm (**A**); 10 µm (**B–D**).

**Figure 5.**
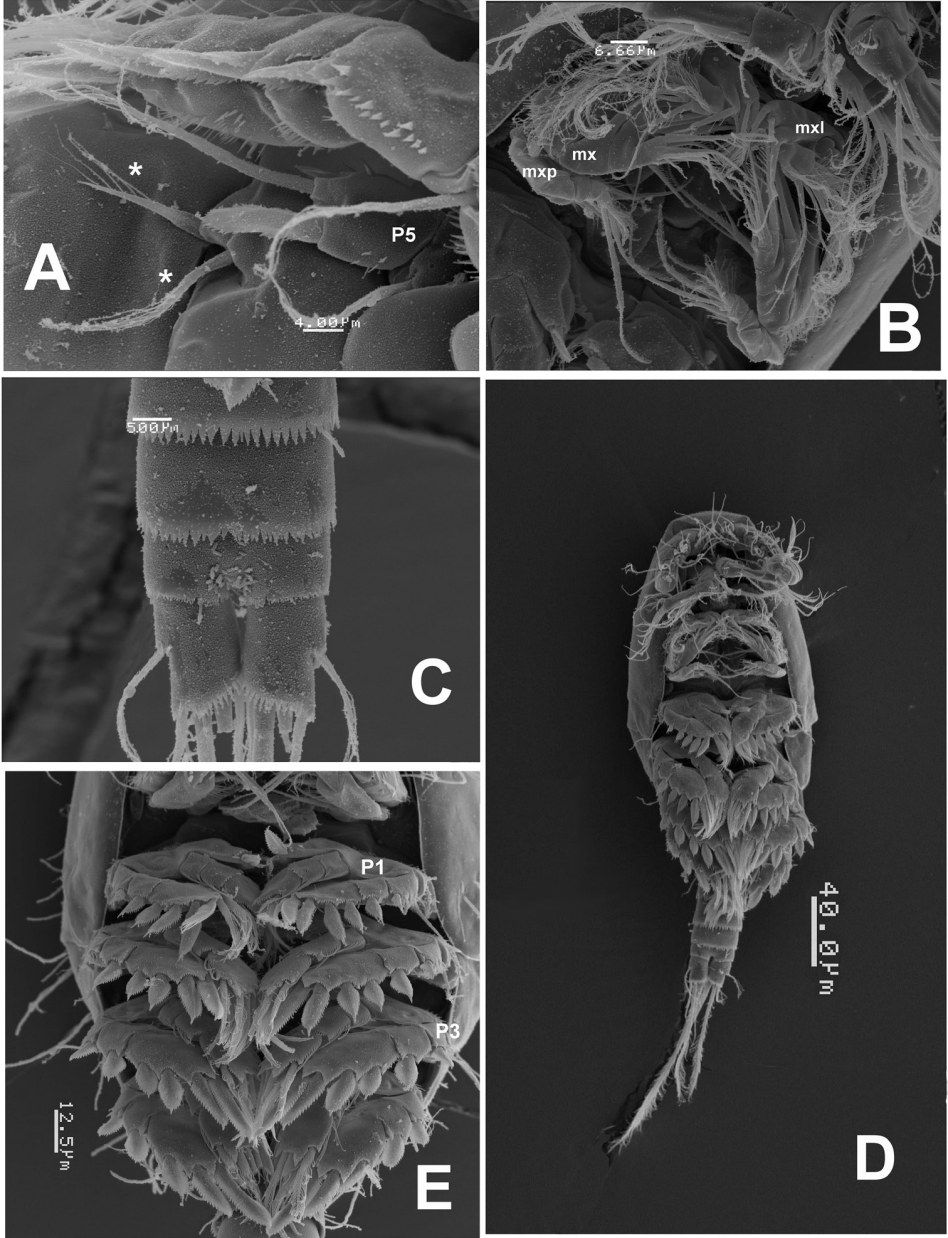
*Mexiclopina campechana* gen. et sp. n., from the Gulf of Mexico, SEM-prepared female. **A** fifth leg and sixth leg armature (indicated by asterisks); male specimen: **B** ventral view of mouthparts including maxillule (mxl), maxilla (mx), and maxilliped (mxp) **C** preanal and anal somites and caudal rami **D** habitus, ventral view **E** legs 1–4 showing ornamentation and part of armature, ventral view; leg 1 (P1) and leg 3 (P3) indicated.

**Figure 6.**
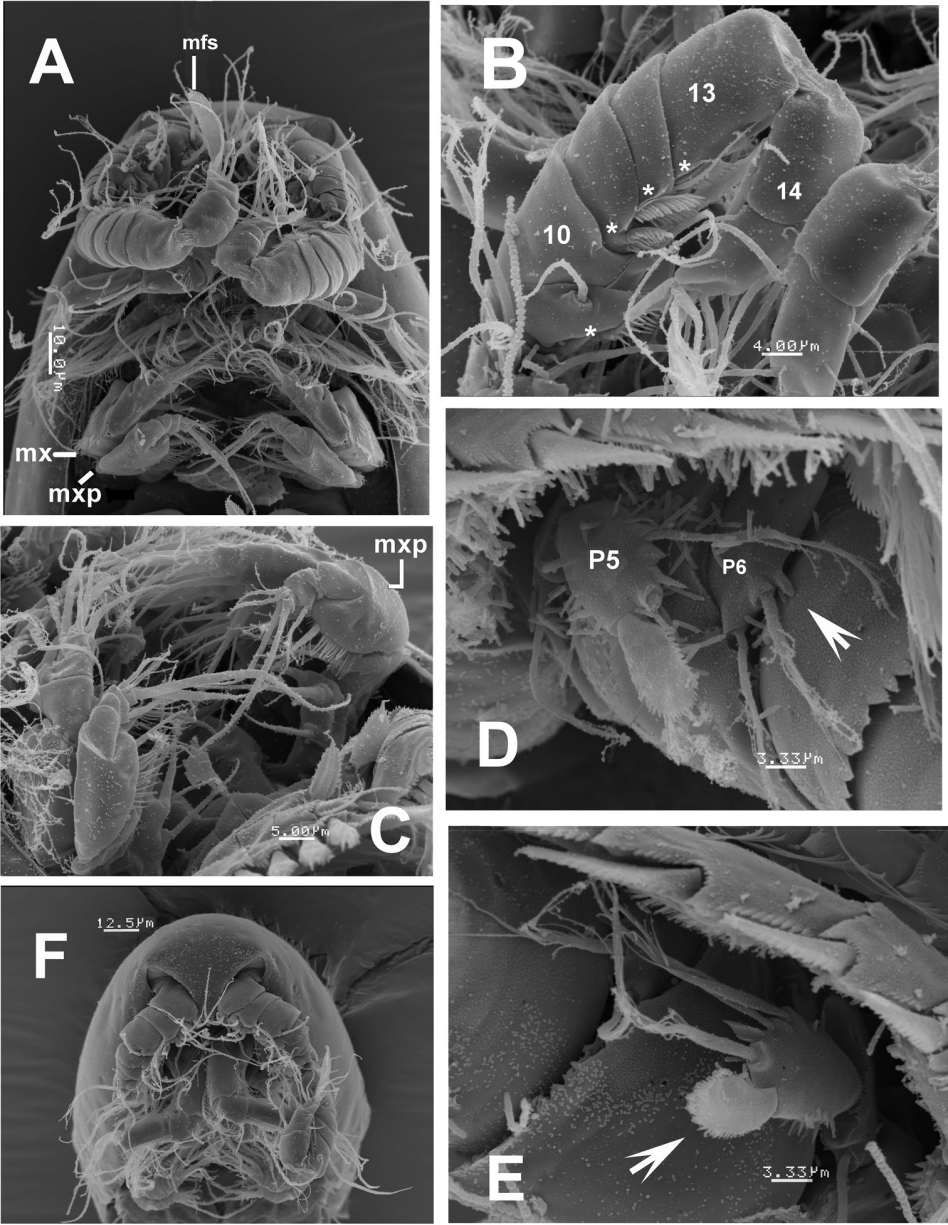
*Mexiclopina campechana* gen. et sp. n., from the Gulf of Mexico, SEM-prepared males. **A** cephalic area showing digeniculate antennules (note flattened aesthetasc-mfs) and mouthparts including maxilla (mx) and maxilliped (mxp) **B** distal segments (9-15) of antennule showing position of pectinate setae (asterisks) **C** detail of maxillipedal (mxp) ornamentation of basis and endopodal segmentation and armature, ventral view **D** fifth leg(P5) partly damaged but with distinctive serrate spine and sixth leg (P6) with inner spiniform process (arrowed) **E** fifth leg **F** rostrum, geniculate antennules and antennae, ventral view; another male specimen.

Figs 1 , 2 , 3 , 4 , 5 , 6

##### Material examined.

Holotype. Adult female, dissected, mounted in glycerin sealed with Entellan (ECO-CHZ-09298), Laguna de Términos, Campeche, Mexico (18°44'29.3"N;91°29'44.6"W), collected February 13, 2015 by R. J. Almeyda-Artigas, C. Lara-Bautista, and C. Chamorro-García. Allotype male, dissected, same site, date, and collectors (ECO-CHZ-09299). Paratypes. Two adult females, dissected, slides (ECO-CHZ-09300), 6 adult females, undissected, ethanol-preserved, vial (ECO-CHZ-09301); 3 adult males, undissected, ethanol-preserved, vial (ECO-CHZ-09302). One female, one male, specimens undissected, ethanol-preserved, same locality and collectors (USNM-1283307). One female and 2 males, all used for SEM analysis. Other material examined included +25 undissected adult and juvenile specimens, deposited at CHUX (G1106, G1107).

##### Description of adult female.

Length range (including caudal rami) of type specimens (*n*=11) 350–400 µm, average: 372 µm. Body cyclopiform (Fig. 1A), robust in dorsal view. Lateral margins of pedigers 3–5 produced posteriorly, with rounded margins. Posterior margins of pedigers 3 and 4 smooth in all specimens examined. Urosome 5-segmented. Posterior margin of urosomites with crenulated hyaline frill (Fig. 3A). Genital double-somite symmetrical (Figs 1A; 3A), broadest at anterior rounded half, slightly tapering posteriorly into straight margins, with pair of dorsal sensilla on posterior margin.

Anal somite with ventral and dorsal surfaces smooth, posterior margin ornamented with row of minute spinules along ventral margin at point of insertion of caudal rami. Anal operculum smooth. Caudal ramus (Fig. 1D) length/width ratio range: 1.17–1.20. Dorsal and ventral surface of caudal rami smooth except for row of spinules along posterior margin at insertion of caudal setae (Fig. 1D). Inner margin of caudal rami smooth. Rami with six setae; seta I absent; seta II inserted midway of outer margin; seta III shorter than seta VI, both lightly plumose; seta IV about 3.2 times as long as seta III, with heteronomous ornamentation, with spinules on proximal outer margin and with plumose distal half; proximal inner margin with few spinules, distal third plumose; seta V longest, about 1.5 times as long as seta IV, naked proximally, with few rigid spinules proximally and lightly plumose distally along both margins; dorsal seta VII as long as seta II, about twice as long as ramus (Fig. 1D). Rostrum wide, tapering distally into pointed tip.

*Antennule* (Fig. 1B): 10-segmented. Surface of segments smooth except for short curved comb of 7-8 spinules placed proximally on first segment. Armature of antennule segments indicating ancestral segmentation (in Roman numerals), with number of setae (Arabic numerals), and aesthetascs (aes) in parentheses: 1(I-II)(3), 2(III-V)(5), 3(VI-IX)(8), 4(X-XI)(4), 5(XII-XIV)(6), 6(XV-XX)(6+ae), 7(XXI-XXII)(2+ae), 8(XXIII-XXV)(3), 9(XXVI)(2), 10(XXVI-XXVIII)(7+ae).

*Antenna* (Fig. 1C): 4-segmented, fused coxa and basis cylindrical, with long lightly setulose basal seta on outer margin and slender, short inner exopodal seta (exp in Fig. 1C). Endopod 3-segmented. First endopodal segment cylindrical, about twice as long as succeeding second segment, with long medial seta reaching distal margin of second endopodal segment; segment ornamented with patch of spinules around insertion of seta. Second endopodal segment with patch of spinules on proximal position. Setation formula of endopodal segments 1-3 as: 1, 5, 6.

*Mandible* (Fig. 2A): with robust gnathobase armed with long setulose seta. Gnathal blade with 7 teeth plus short, uniserially pinnate dorsal seta; row of spinules at base of medial teeth. Basis with long seta plus row of spinules on inner margin. Exopod 4-segmented, armed as 1,1,1,2, surface of segments smooth; apical seta being longest of exopodal setae; distal brush not observed. Fourth segment slightly longer than preceding two exopodal segments. Endopod 2-segmented, setal formula 3, 5; inner margin of proximal segment with row of setules.

*Maxillule* (Figs 2B; 5B): with well-developed precoxal arthrite armed with 9 setae/spines. Coxal epipodite represented by two unequal setae; coxal endite knob-like, armed with long seta. Appendage with two basal endites, proximal with three, distal with two setae. Endopod rounded, unsegmented, armed with 7 setae; exopod subrectangular, unsegmented, with 4 apical, relatively short stout setae (asterisks in Fig. 2B).

*Maxilla* (Fig. 2C): 5-segmented, syncoxal endites with setal formula as 3,1,3,3. Basis with robust claw and two pinnate setae; endopod 3-segmented, first and second segments with three and two setae, respectively, third with 4.

*Maxilliped* (Figs 2D; 5B): slender, 6-segmented, precoxa and coxa fused forming syncoxal segment with three endites; proximal endite with single seta, second with 3 unequal setae, third endite ornamented with cluster of cuticular scales, armed with two long, subequal stout setae. Basis expanded distally, medial margin ornamented with row of long, stiff setules, and with two subdistal setae. Endopod 4-segmented; first and second segments naked, third segment with one lightly plumose seta, fourth segment with four elements including two short plumose and two long, stout simple setae.

*Legs 1–4* (Fig. 3B-H): biramous, each with distinct coxa and basis and 3-segmented endopodal and exopodal rami. Outer margin of all segments finely serrate in both sexes (Fig. 5E). Spines on exopodal segments flanged with serrate hyaline frill.

*Leg 1* (Fig. 3B, C): intercoxal sclerite subrectangular, with two medial spiniform processes on distal margin (Fig. 3F), otherwise smooth. Coxa with two submarginal short rows of minute spinules on outer margin and with pinnate inner seta. Basipod with long flexible outer seta almost reaching distal margin of exopodal ramus, plus stout, robust flanged inner spine (asterisk in Fig. 3B).

*Legs 2–3*: each with exopodal ramus longer than endopod, intercoxal sclerites with distal margin smooth (Fig. 3G). Coxa with two rows of spinules on outer margin; insertion point of inner seta naked. Basipod with outer seta shorter than leg 1 counterpart (Fig. 3D).

*Leg 4* (Fig. 3E): posterior surface of coxa furnished with two rows of minute spinules on proximal and lateral margins. Intercoxal sclerite posterior margin smooth (Fig. 3H). Basipod with outer seta shorter than leg 1 counterpart (Fig. 3E). Third endopodal segment with outermost subdistal setal element modified, proximal half stouter, wider than flexible, whip-like distal half (asterisk in Fig. 3E).

Armature formula of swimming legs as:

*Leg 5* (Figs 2E; 3A): with coxobasis subrectangular, armed with single seta on outer margin, inner margin smooth. Exopod unsegmented, subrectangular, ornamented with longitudinal row of few spinules along inner margin and group of minute spinules on outer margin (Figs 2E; 5A). Exopod armature consisting of one short inner spine, one medial setulose seta and one outer blunt spine flanged with serrate hyaline frill, latter about 2.4 times as long as inner spine.

*Leg 6* (Fig. 5A): inserted laterally, represented by short plate armed with inner slender unipinnate spine and outer setulose seta (asterisks in Fig. 5A).

##### Description of adult male.

Length of allotype 325 µm, of rest of male paratypes (*n*=7): 313–328 µm, average 321 µm. Body cyclopiform, smaller than female and slightly narrower (Figs 4A; 5D). Rostrum as in female (Fig. 6F). Length/width ratio of caudal ramus 1.20–1.22, setation pattern as in female (Figs 4B; 5C). Antennules, symmetrical, digeniculate, 15-segmented (Figs 4C; 6A, B). Segment 9 concave, partially covering proximal half of succeeding segment 10. Armature of segments as follows: 1(I-II)(2), 2(III-V)(6), 3(VI-VIII)(3), 4(IX)(1+ae), 5(X-XI)(1), 6(XII)(naked), 7(XIII)(2), 8(XIV)(2), 9(XV)(1+sp), 10(XVI)(2+sp), 11(XVII)(sp), 12(XVIII)(1+sp), 13(XIX-XX)(1+sp), 14(XXI-XXII)(1+sp), 15(XXIII-XXVIII)(11+2ae). Geniculations between ancestral segments XV and XVI (9–10) and XX-XXI (13–14).Spines on segments 9–12 pectinate (asterisks in Fig. 6B). Terminal segment with modified, hypertrophied flattened aesthetasc on apical position (mfs in Fig. 6A). Segmentation and setation pattern of mouthparts (Figs 6A, C, F; 5B) and swimming legs 1-4 (Fig. 5D, E) as in female.

*Leg 5* (Figs 4D; 6D, E) with coxobasis subrectangular, armed with outer seta. Exopod unsegmented, ornamented with few spinules on inner margin and group of minute spinules on outer margin (Figs 4D, 6E). Exopod armed with five elements, two long, inner setae, one small medial spine, one medial seta and outer flanged spine with serrate hyaline frill; as in female, latter element (arrowed in Fig. 6E) blunt, about 2.5 times as long as inner spine.

*Leg 6* represented by flat, rounded plate bearing two slender setae and an inner spiniform process (Figs 4D; 6D).

##### Type locality.

Laguna de Términos (18°44'29.3"N; 91°29'44.6"W), state of Campeche, Mexico, southern Gulf of Mexico.

##### Etymology.

The species is named after the state of Campeche in southeast Mexico. Gender is feminine.

##### Habitat.

The lagoon has a length of 70 km and 30 km at its widest sector. It has extense coverage of seagrass beds (mainly *Thalassia testudinum*), mangrove areas and zones with no vegetation. It is a shallow system, (average depth = 2.5 m). The lagoon receives freshwater input from several rivers. Most of its bottom is covered by sediments of sand, silt and clay with a high content of calcium carbonate mainly in the vicinity of Boca de Puerto Real (between 50 and 70%).

##### Remarks.

Based on the first examination of these specimens, they were tentatively identified as a species of *Cyclopina* Claus, 1863 by the combined display of the following features: 10-segmented female antennule with sixth antennulary segment being longest, antenna with single exopodal seta; female fifth leg exopod with three armature elements, the apical seta flanked by two spines; leg 1 with 3-segmented endopod; and caudal seta I absent (cf. Vervoort 1964; Jaume and Boxshall 1997; Boxshall and Halsey 2004). However, when Jaume and Boxshall’s (1996b) key to the cyclopinid genera was run, our specimens could not be adequately placed in a genus and it did not fit in the generic diagnoses of other related cyclopinids (Jaume and Boxshall 1996a, b, 1997; Martínez Arbizu 1997a, b; Humes 1999; Ivanenko and Defaye 2004). Also, based on our morphological comparison with *Cyclopina esilis* Brian, 1938, the best described species of *Cyclopina* (Jaume and Boxshall 1996a), it was clear that despite their affinities, the new genus and *Cyclopina* diverge in several important characters. In addition, the monotypic genus *Heptnerina* (Ivanenko and Defaye 2004) shares some characters with the new genus (i.e., swimming legs segmentation, number of female antennulary segments, armature of male and female fifth legs, segmentation of mandible palp) but differ in some others, as explained below. Overall, the genus *Mexiclopina* gen. n. differs from the other cyclopinid genera in having a unique combination of characters including: 1) absence of modified brush-like seta on the 4th mandibular exopodal segment; 2) maxillule exopod with stout setal elements and no brush-like setae; 3) presence of modified seta on the fourth leg endopod; 4) fifth leg exopod armed with three elements in the female and five in the male; 5) outer exopodal spine of leg 5 blunt in both sexes; 6) male sixth leg with two outer slender setae and inner spiniform process; 7) intercoxal sclerite of first swimming leg with two medial spiniform processes on distal margin. The new genus diverges from *Heptnerina* in the lack of an endopodal lobe in leg 5, in the presence of a single antennary exopodal seta *vs.* two setae present in *Heptnerina confusa* (Ivanenko and Defaye 2004, fig. 3A), and the lack of a modified seta on the maxillule exopodal lobe and also in the mandible exopod (Ivanenko and Defaye 2004, figs. 3A, C). The new genus differs from *Cyclopina* in the lack of a brush-like seta on the mandible exopod (Jaume and Boxshall 1996a; Lotufo 1994); this character is distinctive of the genus and it is present in the type species, *Cyclopina gracilis* Claus, 1863. Remarkably, in the new genus the intercoxal sclerite of leg 1 has a distinctive feature not previously observed in *Cyclopina*; it has two medial spiniform processes on the posterior margin (Fig. 3F), similar acute processes in leg 1 are present in *Troglocyclopina balearica* Jaume & Boxshall, 1996 (Jaume and Boxshall 1996b), but are absent in *Heptnerina* (Ivanenko and Defaye 2004). The new genus clearly diverges from *Troglocyclopina* Jaume & Boxshall, 1996 in having six setae instead of five on the distal segment of endopod of leg 1 (Jaume and Boxshall 1996b, figs. 4A) but also in the presence of two exopodal setae on the antenna (Jaume and Boxshall 1996b, fig. 3A) *vs.* a single exopodal seta in *Mexiclopina*.

Other remarkable features of the new genus include: 1) the short, stout distal setae of the exopodal segment of the maxillule (asterisks in Fig. 2B); these setae are long, flexible in *Cyclopina* (Lotufo 1994; Jaume and Boxshall 1996a; Karanovic 2008) and *Heptnerina* (Ivanenko and Defaye 2004); 2) the female P6, represented by short plate armed with two slender setae; it is similar to that known in species of *Cyclopina* but differs from *Heptnerina* (Ivanenko and Defaye 2004, fig. 1E), with three unequal setae; and 3) the modified, short spiniform outer seta of the third endopodal segment of leg 4 (asterisk in Fig. 3E), not described in any other cyclopinid.

Because of the close morphological resemblance of the new species with *Cyclopina*, we performed a comparison with the most closely related species of this genus. Only a few species of *Cyclopina* have a female leg5 with the inner spine of the exopodal segment less than half the length of the outer spine, the latter being longer than the segment itself (Jaume and Boxshall 1996a). This group of species include *Cyclopina kieferi* Schäfer, 1936, from Europe, *Cyclopina esilis* Brian, 1938 from Mediterranean anchialine caves, *Cyclopina americana* Herbst, 1982, from North Carolina, USA, *Cyclopina caissara* Lotufo, 1994 from Brazil (Lotufo 1994) and from the Mexican Pacific (Gómez and Martínez Arbizu 2004), and *Cyclopina amita* from Australia (Karanovic 2008). The new species shares this feature with this group of species but it can be easily distinguished from *Cyclopina caissara* by the segmentation of the antennules, the new species having 10 segments, like most other known species of *Cyclopina*, whereas *Cyclopina caissara* has a 12-segmented antennule both in specimens from Brazil (Lotufo 1994, fig. 37) and from Mexico (Gómez and Martínez Arbizu 2004, fig. 3A). Also, the length/width ratio of the caudal rami differs between these two species, being slightly longer in *Cyclopina caissara* (ratio=1.3–1.5; Lotufo 1994; Gómez and Martínez Arbizu 2004) *vs.* 1.17–1.2 in the new species. The shape and size of the outermost terminal flanged spine of the male fifth leg differ in these species, being broad and blunt in the new species *vs.* slender and pointed in *Cyclopina caissara* (Lotufo 1994, fig. 52). Also, the female fifth leg differs in the size and proportions of these spines; the outer spine is more than 4 times as long as the inner one in *Cyclopina caissara* (Lotufo 1994, fig. 49), whereas in the new species this element is only about twice longer than the inner spine. In *Cyclopina caissara* the armature of the female sixth leg consists only of two elements, the inner one corresponding to a thick stout serrate seta (Lotufo 1994, fig. 50; Gómez and Martínez Arbizu 2004, fig. 1C), thus differing from the slender seta present in homologous position in the new species (Fig. 5A).

The new species differs from *Cyclopina esilis* in the display of a long terminal seta on the exopod of mandibular palp; it is the longest and is slightly broader than the rest of exopodal setae; contrastingly, this seta is remarkably short and modified, umbrella-like, in *Cyclopina esilis* (Jaume and Boxshall 1996a, fig. 2B). In addition, both species can be readily distinguished by the proportions of the caudal rami, being 2.6–3.3 times longer than wide, relatively elongate in *Cyclopina esilis* (Jaume and Boxshall 1996a, fig. 1F,G), *vs.* short and subquadrate (length/width ratio 1.2) in the new species.

*Mexiclopina campechana* sp. n. differs from *Cyclopina americana* in body shape, with the third and fourth pedigerous somites strongly produced posteriorly, the process of the fourth somite reaching well beyond the posterior margin of the fifth pedigerous somite (Fig. 1A); in *Cyclopina americana* the posterolateral corners of the fourth pedigerous somite do not reach the posterior margin of the succeeding somite (Herbst 1982, fig. 1). Also, in *Cyclopina americana* the female anal somite is 1.16 times as long as the caudal ramus (Herbst 1982, fig.1), whereas in the new species the anal somite is shorter (0.8 times) than the caudal ramus. The length/width ratio of the caudal rami is also slightly different in both species, 1.2 in *Mexiclopina campechana* sp. n., *vs.* 1.3 in *Cyclopina americana* (Herbst 1982, fig. 2). They differ also in the relative length of the antennulary segments, particularly in the shorter segment 6 in *Cyclopina americana*, which is 26% of the antennule length (Herbst 1982, fig. 3), *vs.* 21% in the new species from Campeche. In *Cyclopina americana* the antenna lacks the exopodal seta (Herbst 1982, fig. 4), which is present in the new species (Fig. 1C), but in some species like *Cyclopina amita* this seta is also absent (Karanovic 2008). In ventral view the male anal somite of *Cyclopina americana* is long, 1.45 times as long as the caudal rami (Herbst 1982, fig. 10), whereas in the new species it is relatively shorter, 0.7 times as long as the caudal ramus (Figs 4A; 5C). In addition, both sexes have a crenulate hyaline frill on the posterior margin of urosomites(Figs 3A, 5C), whereas these margins are smooth in both sexes in *Cyclopina americana* (Herbst 1982, figs 1;10; 11). In *Cyclopina americana* the male fifth leg has four elements on the exopodal segment (Herbst 1982, fig. 13), *vs.* five in the new species. In addition, the sixth leg of the new species has, like the majority of the species of *Cyclopina* for which males are known (Karanovic 2008), an inner spine aside the two usual setae; this spine is absent in both *Cyclopina americana* (Herbst 1982, figs 10;11) and *Cyclopina amita* (Karanovic 2008, fig. 36C). The new species differs from *Cyclopina amita* in the antennule segmentation; this appendage having 11 segments in the Australian species (Karanovic 2008, fig. 34A) *vs.* 10 segments in *Mexiclopina campechana*.

The new species of *Mexiclopina* shows also some resemblance with *Cyclopina kieferi*, but in this species the external spine of the female fifth leg is 1.2–1.5 times as long as the internal spine (*vs.* 2.5 in the new species), the caudal rami are clearly longer than the anal somite and have a length/width ratio of 2.6 (Vervoort 1964; Lotufo 1994),thus differing from *Mexiclopina campechana*, with an anal somite as long as the caudal rami, which in turn have a 1.2 length/width ratio.

Males are known for only about half the known nominal species of *Cyclopina* (Karanovic 2008) and the available keys are based on females (Vervoort 1964), thus, characters of this gender have not been fully explored but some of them appear to be potentially important to define species. For instance, the male of *Cyclopina esilis* shares several features with the new species, but the antennulary armature differs. The male antennule of *Cyclopina esilis* has pectinate spines on each of segments 10-13 (Jaume and Boxshall 1996, fig. 4D), whereas these spines are distributed on segments 9-12 in the new species (Fig. 6B). In addition, the male antennule of *Cyclopina americana* has 13 segments (Herbst 1982, fig. 12) *vs.* 15 in the new species; the last antennular segment is distinctly acute in *Cyclopina americana* (Herbst 1982, fig. 12) and blunt in the new species. Details of the male antennulary armature were not shown in the description of *Cyclopina americana* (Herbst 1982), but this appendage is likely to provide additional differences at the species level.

The male fifth leg of the new species has 5 elements on the exopodal segment, thus diverging from most species of *Cyclopina* for which males have been described thus far. This feature is shared only with *Cyclopina esilis*, *Cyclopina caissara*, *Cyclopina kieferi*, *Cyclopina amita*, and *Cyclopina confusa*, but the latter has an ornamented anterior surface of the female fifth leg, thus diverging from the smooth condition of the same surface in *Mexiclopina campechana*.

The copepod fauna of the Laguna de Términos has been known mainly from plankton surveys (Suárez-Caabro and Gómez-Aguirre 1965; Salas-Marmolejo 1981); relatively little is known from other copepod habitats. The local copepod diversity of interstitial environments may equal or exceed that of their planktonic relatives. The sampling of shallow coastal systems frequently results in the capture of epibenthic or interstitial fauna that is integrated into the water column. This appears to be the case in the new species, belonging to a genus of interstitial forms (Karanovic 2008).

This work increases the number of species of cyclopinids known from the Americas (Wilson 1932; Nicholls 1939; Herbst 1982; Reid 1990; Lotufo and Rocha 1991; Lotufo 1994; Rocha and Botelho 1998; Gómez and Martínez Arbizu 2004). Records of this family now comprise thirteen species of *Cyclopina*: *Cyclopina agilis* Wilson, 1932, *Cyclopina laurentica* Nicholls, 1939, *Cyclopina vachoni* Nicholls, 1939, *Cyclopina americana* Herbst, 1982, *Cyclopina caiala* Lotufo & Rocha, 1991, *Cuipora janaina* (Lotufo & Rocha, 1991), *Cyclopina arenosa* Lotufo, 1994, *Cyclopina caissara* Lotufo, 1994, *Cyclopina caroli* Lotufo, 1994, *Cyclopina mediterranea* Steuer, 1940, *Cyclopina dorae* Lotufo, 1994, *Cyclopina yutimaete* Lotufo, 1994, and a species of the new genus, *Mexiclopina campechana*. The new species is the first cyclopinid described from Mexico, and represents the first record of the family in the Gulf of Mexico (see Suárez-Morales et al. 2009). After the finding of *Cyclopina caissara* in the Mexican Pacific coast (Gómez and Martínez Arbizu 2004), it is the second record of cyclopinids in the country.

## Supplementary Material

XML Treatment for Mexiclopina

XML Treatment for Mexiclopina campechana

## References

[B1] Boxshall GA (2015) *Cyclopina* Claus, 1863. In: Walter TC Boxshall G (2015) World of Copepods database. World Register of Marine Species. http://www.marinespecies.org/aphia.php?p=taxdetails&id=106437 [accessed on 2015-03-27]

[B2] Boxshall GA Halsey SH (2004) An Introduction to Copepod Diversity. The Ray Society, London, 966 pp.

[B3] Brian A (1938) Description d’une nouvelle espèce de Copépode Cyclopoide du genre *Cyclopina* (*C. esilis* n. sp.). Bulletin de la Société Zoologique de France 63: 13–18.

[B4] Claus C (1863) Die freilebenden Copepoden mit besonderer Berücksichtigung der Fauna Deutschlands, der Nordsee, und des Mittelmeeres. Verlag von Wilhelm Engelmann, Leipzig, 230 pp. doi: 10.5962/bhl.title.58676

[B5] Gómez S Martínez Arbizu P (2004) First record of the genus *Cyclopina* (Copepoda: Cyclopoida), and fully illustrated redescription of *Cyclopina caissara* from northwestern Mexico. Anales del Instituto de Biología, Universidad Nacional Autónoma de México, Serie Zoología 75: 121–134.

[B6] Herbst HV (1982) Drei neue marine Cyclopoida Gnathostoma (Crustacea: Copepoda) aus dem nordamerikanischen Kiistenbereich. Gewässer und Abwasser 68/69: 107–124.

[B7] Humes AG (1999) Copepoda (Cyclopinidae and Misophriidae) from a deep-sea hydrothermal site in the northeastern Pacific. Journal of Natural History 33: 961–978. doi: 10.1080/002229399300038

[B8] Ivanenko VN Defaye D (2004) A new genus and species of deep-sea cyclopoids (Copepoda, Cyclopinidae) from the Mid-Atlantic Ridge (Azores Triple Junction, Lucky Strike). Zoosystema 26(1): 49–64.

[B9] Jaume D Boxshall GA (1996a) Rare cyclopoid copepods (Crustacea) from Mediterranean littoral caves. Bulletin of the Natural History Museum Zoology Series 62: 83–99.

[B10] Jaume D Boxshall GA (1996b) Two new genera of cyclopinid copepods (Crustacea) from anchialine caves on western Mediterranean and eastern Atlantic islands. Zoological Journal of the Linnean Society 117: 283–304. doi: 10.1111/j.1096-3642.1996.tb02191.x

[B11] Jaume D Boxshall GA (1997) Two new genera of cyclopinid copepods (Cyclopoida: Cyclopinidae) from anchialine caves of the Canary and Balearic Islands, with a key to genera of the family. Zoological Journal of the Linnean Society 120: 79–110. doi: 10.1111/j.1096-3642.1997.tb01273.x

[B12] Karanovic T (2008) Marine interstitial Poecilostomatoida and Cyclopoida (Copepoda) of Australia. Crustaceana Monographs 9 Koninklijke Brill, Leiden, 336 pp. doi: 10.1163/ej.9789004164598.i-332

[B13] Lotufo GR (1994) *Cyclopina* (Copepoda, Cyclopoida) from Brazilian sandy beaches. Zoologica Scripta 23: 147–159. doi: 10.1111/j.1463-6409.1994.tb00381.x

[B14] Lotufo GR Rocha CEF (1991) Copepods from intertidal interstitial water of Salvador, Brazil. I. *Cuipora janaina* gen. n., sp. n. and *Cyclopina caiala* sp. n.(Cyclopoida: Cyclopinidae). Bijdragen tot de Dierkunde 61: 107–118.

[B15] Martínez Arbizu P (1997a) *Cyclopicina sirenkoi* sp. n. (Copepoda: Cyclopinidae) from deep waters in the Laptev Sea (Arctic Ocean), with comments on the phylogenetic relationships of copepod orders. Senckenbergiana Biologica 77: 89–99.

[B16] Martínez Arbizu P (1997b) A new genus of cyclopinid copepods (Crustacea), with a redescription of *Smirnovipina barentsiana* comb. nov. (Smirnov,1931). Sarsia 82: 313–323.

[B17] Nicholls AG (1939) Marine harpacticoids and cyclopoids from the shores of the St. Laurence. Station Biologique de Saint-Laurent. Fauna et Flora Laurentianae. 2. Naturaliste Canadien 66: 241–316.

[B18] Ramos-Miranda J Flores-Hernández D Ayala-Pérez LA Rendón-Von Osten J Villalobos G Sosa-López A (2006) Atlas hidrológico e ictiológico de la Laguna de Términos. EPOMEX Universidad de Campeche/CONANP, 173 pp.

[B19] Reid JW (1990) Continental and coastal free-living Copepoda (Crustacea) of Mexico, Central America and the Caribbean region. In: Navarro D Robinson JG (Eds) Diversidad Biológica en la Reserva de la Biosfera de Sian Ka’an, Quintana Roo, México. CIQRO/Univ. of Florida, Mexico City, 175–213.

[B20] Reid JW (2003) A technique for observing copepods. In: Ueda H Reid JW (Eds) CopepodaCyclopoida. Genera *Mesocyclops* and *Thermocyclops*. Guides to the Identification of the Microinvertebrates of the Continental Waters of the World. 20 Backhuys Publishers, Amsterdam, 8.

[B21] Rocha CEF Botelho MJC (1998) Maxillopoda-Copepoda. Cyclopoida. In: Young PS (Ed.) Catalogue of Crustacea of Brazil (Série Livros N. 6). Museu Nacional, Rio de Janeiro, 129–166.

[B22] Salas-Marmolejo J (1981) Distribución y abundancia de los copépodos (Copepoda) en la Laguna de Términos, México, durante un ciclo anual (1978). B.Sc. Thesis, Fac. Ciencias, Universidad Nacional Autónoma de México.

[B23] Suárez-Caabro JA Gómez-Aguirre S (1965) Observaciones sobre el plancton de la Laguna de Términos, Campeche, México. Bulletin of Marine Science 15: 1072–1120.

[B24] Suárez-Morales E Fleeger JM Montagna PA (2009) Free-living Copepoda of the Gulf of Mexico. In: Felder DL Camp DK (Eds) Gulf of Mexico – Its Origins, Waters, and Biota, Biodiversity. Texas A&M University Press, 841–870.

[B25] Steuer A (1940) Über einige Copepoda Cyclopoida der Mediterranen Amphioxussande. Note dell’Istituto Italo Germanico di Biologia Marina di Rovigno d’Istria 2(17): 1–27.

[B26] Vervoort W (1964) Free-living Copepoda from Ifaluk Atoll in the Caroline Islands. United States National Museum Bulletin 236: 1–431. doi: 10.5479/si.03629236.236.1

[B27] Walter TC Boxshall GA (2015) Cyclopinidae Sars G.O., 1913. In: Walter TC Boxshall GA (2015) World of Copepods database. World Register of Marine Species. http://www.marinespecies.org/aphia.php?p=taxdetails&id=106414 [accessed on 2015-09-02]

[B28] Wilson CB (1932) The copepods of the Woods Hole region, Massachusetts. Bulletin of the United States National Museum 158: 1–635. doi: 10.5479/si.00963801.80-2915.1

[B29] Yáñez-Arancibia A Day Jr JW (1982) Coastal Lagoons. Ecological characterization of Terminos Lagoon, a tropical lagoon-estuarine system in the Southern Gulf of Mexico. Oceanologica Acta Special 5: 431–440.

